# Nutritional Deficiencies and Management in Tuberculosis: Pharmacotherapeutic and Clinical Implications

**DOI:** 10.3390/nu17111878

**Published:** 2025-05-30

**Authors:** Anca Ionela Fâcă, Denisa Ioana Udeanu, Andreea Letiția Arsene, Beatrice Mahler, Doina Drăgănescu, Miruna-Maria Apetroaei

**Affiliations:** 1 Faculty of Pharmacy, Carol Davila University of Medicine and Pharmacy, 6, Traian Vuia Street, 020956 Bucharest, Romania; anca-ionela.faca@drd.umfcd.ro (A.I.F.); andreea.arsene@umfcd.ro (A.L.A.); doina.draganescu@umfcd.ro (D.D.); miruna-maria.apetroaei@rez.umfcd.ro (M.-M.A.); 2 Marius Nasta Institute of Pneumology, 90, Viilor Street, 050159 Bucharest, Romania; beatrice.mahler@umfcd.ro; 3 Faculty of Medicine, Carol Davila University of Medicine and Pharmacy, 8, Eroii Sanitari Street, 050474 Bucharest, Romania

**Keywords:** tuberculosis management, nutritional support, drug–food interactions, antituberculosis therapy, tuberculosis deficiencies, clinical nutrition

## Abstract

Tuberculosis is an infectious condition caused by *Mycobacterium tuberculosis*, primarily targeting the pulmonary system, with the potential to disseminate to various other organs via the haematogenous pathway, ranking among the top ten causes of global mortality. Tuberculosis remains a serious public health problem worldwide. This narrative review aims to emphasise the clinical importance of the inter-relationships between nutrition, pharmacotherapy, and the most common drug–nutrient interactions in the context of tuberculosis and multi-drug-resistant tuberculosis management. Nowadays, pharmacologic approaches utilise polytherapeutic regimens that, although showing increased efficacy, prominently affect the nutritional status of patients and modify multiple metabolic pathways, thus influencing both the effectiveness of therapy and the patient outcomes. There is much evidence that antituberculosis drugs are associated with deficiencies in essential vitamins and various micronutrients, leading to serious adverse consequences. Moreover, poor nutrition exacerbates TB outcomes, and TB further exacerbates nutritional status, a vicious cycle that is particularly prevalent in low-resource environments. Nutritional support is necessary, and clinicians ought to evaluate it on a patient-by-patient basis, as empirical evidence has shown that it can improve immune recovery, decrease tuberculosis-associated morbidity, and increase adherence to therapy. However, drug–food interactions are increasingly prevalent, and patients with tuberculosis require personalised dietary and pharmacological regimens. In this context, antituberculosis treatment requires a holistic approach, based on the collaboration of the prescribing physician, pharmacist, and nutritionist, to assess the patient’s needs from a nutritional and pharmacological perspective, with the ultimate goal of decreasing mortality and improving the prognosis of patients through personalised therapies.

## 1. Introduction

*Mycobacterium tuberculosis* is the causative agent of tuberculosis (TB), a contagious infectious disease that typically persists throughout life and causes tubercles to form in various body areas. Roughly two billion individuals worldwide are currently infected with *Mycobacterium tuberculosis*, which has very ancient beginnings. With new cases of TB occurring each year, nearly one-third of the world’s population is at risk of contracting the active disease due to being carriers of the TB bacillus [1]. According to estimates, 10.8 million individuals worldwide contracted TB in 2023, which is approximately 134 incident cases per 100,000 people. TB is still one of the most significant causes of mortality globally. People with HIV accounted for 6.1% of all incident TB cases. The WHO areas of South-East Asia (45%), Africa (24%), and the Western Pacific (17%) had the highest percentages of TB cases in 2023. The Eastern Mediterranean (8.6%), the Americas (3.2%), and Europe (2.1%) had lower percentages [2]. Its projected annual incidence and mortality rate highlight the disease’s healthcare and economic cost. The WHO has developed and suggested a number of international strategies to combat TB; however, the anticipated decrease in TB incidence has been delayed by the failure of national and local governments to implement WHO strategies, as well as by the growing epidemic of HIV/TB co-infection and multidrug-resistant (MDR) TB [3].

It is still challenging to meet the World Health Organisation’s End TB Strategy goals of 90% TB incidence and 95% TB mortality reduction globally by 2035. Global TB control continues to be challenged by the ongoing expansion of MDR-TB [4]. Nonsusceptibility to at least one agent in at least three antimicrobial categories is referred to as MDR; extensively drug-resistant (XDR) as susceptibility to at most two categories; and pan drug resistance (PDR) as nonsusceptibility to all drugs across all antimicrobial categories [5,6]. Following a gradual decline from 2015 to 2019, the predicted yearly number of individuals who contracted MDR/RR-TB was comparatively consistent worldwide between 2020 and 2023. In 2023, that figure was projected to be 400,000 [2]. The poor identification rate, high treatment failure rate, and high rate of mortality of MDR/XDR-TB make its widespread distribution concerning. The primary cause of the rising number of MDR/XDR-TB cases is the inability of diagnostic tests to identify the pathogen’s drug susceptibility, which aids in the disease’s transmission from person to person. Furthermore, the eventual development of medication resistance may also result from inadequate drug therapy or inappropriate drug regimens [7,8].

*Mycobacterium tuberculosis* has developed the ability to thrive by exploiting the human immune system to enter the host and reside within it for an extended period. *M. tuberculosis* is a non-motile, intracellular pathogenic bacterium that divides its cells once every 18 to 24 h and contains a covering of mycolic acid. The bacteria and humans have coevolved for a long time, and special antibacterial defences have evolved that allow them to remain inside the host. Aerosolization, macrophage phagocytosis, phagolysosome blockage and replication, T helper type 1 response, granuloma formation, clinical manifestations, and transmission are the seven relative steps in active *M. tuberculosis* infection, tuberculosis pathophysiology, and disease transmission [9,10]. The diagnosis of tuberculosis is typically made when a patient with symptoms seeks medical attention. In certain cases, such as when trauma is assessed with a chest radiograph that unexpectedly shows TB in the lungs, TB disease can be discovered while the patient is being treated for an unrelated issue. In other cases, a TB contact examination may reveal the presence of TB disease following a recent infection. TB disease typically develops gradually. An immunological deficit may be the cause of a quick onset, which is uncommon [11].

Malnutrition is a significant health issue in less developed areas because it is connected to higher rates of disease susceptibility and mortality, which are typically linked to an elevated incidence of bacterial and parasite infections [12]. Cell-mediated immunity impairment, decreased circulating T-lymphocytes, especially CD4+ helper T-cells and CD3+ CD25+ T-cells with the IL-2 receptor, decreased lymphocyte stimulation reaction to mitogens and antigens, altered cytokine production, decreased secretory immunoglobulin A antibody response on mucosal surfaces, decreased antibody affinity, and phagocyte dysfunction have been documented to be linked to nutritional deficiencies [13].

This narrative review aims to highlight the need for an integrated dietary and pharmaceutical care tailored to each patient’s particular needs in order to maximise clinical results, given the primary challenges posed by tuberculosis and its MDR forms. This research provides an updated overview of the common interactions between antituberculosis medications and nutrients, emphasising how these interactions affect patient nutritional status and therapeutic efficacy. Pharmacological, nutritional, and clinical practice concerns are all combined in an integrative approach to TB patient management, which enables individualised treatment and supports a favourable prognosis.

## 2. Current Pharmacotherapy Guidelines in Tuberculosis

### 2.1. First-Line Antituberculosis Drugs

In addition to other public health measures such as isolation and cough etiquette, treating tuberculosis with antibiotics can help reduce its spread to other people. Treatment for tuberculosis typically consists of two stages: the continued (sterilising) phase and the intensive (bactericidal) phase. Semi-dormant bacteria are eradicated in the continuation phase, whereas mycobacteria with a high rate of replication are killed during the intensive phase [3,14]. Early TB treatment lowers the severity of the illness, the chance of transmission, and the emergence of drug resistance. The recommended treatment for drug-susceptible TB includes a two-month intensive phase of isoniazid, rifampicin, pyrazinamide, and ethambutol, followed by a four-month continuation phase of isoniazid and rifampin [15]. Rifampicin, ethambutol, pyrazinamide, and levofloxacin therapy for six months is the suggested backup regimen for treating confirmed isoniazid-resistant but rifampicin-susceptible tuberculosis. When isoniazid resistance is established and the use of streptomycin or other injectable medications is not advised, this regimen is utilised exclusively. The backup plan is a six-month course of isoniazid, rifampicin, pyrazinamide, and ethambutol if resistance or intolerance prevents the use of a fluoroquinolone, such as levofloxacin [16].

The mechanism of action of isoniazid is complex and encompasses multiple pathways related to macromolecular synthesis that are influenced, particularly the synthesis of mycolic acid. The pro-drug isoniazid undergoes activation via catalase-peroxidase, resulting in active products that interact with enzymes such as enoyl acyl carrier protein reductase and beta-ketoacyl ACP synthase [17]. The action mechanism of rifampicin involves inhibiting DNA-directed RNA synthesis in *Mycobacterium tuberculosis* through interaction with the β subunit of RNA polymerase [18]. Pyrazinamide’s in vivo sterilising action can be explained by its ability to enter all types of TB lung lesions, where it targets nongrowing, drug-tolerant *Mycobacterium tuberculosis*. By attaching to aspartate decarboxylase and preventing this enzymatic process, pyrazinoic acid, the bioactive molecule, prevents bacteria from synthesising Coenzyme A [19]. Ethambutol is regarded as a bacteriostatic drug because it prevents bacilli from proliferating by interfering with the cell wall’s production of arabinogalactan. The *inhA* gene, which is a targeted gene by isoniazid and encodes for an enoyl-acyl carrier protein reductase, is believed to have synergistic effects with ethambutol against *Mycobacterium tuberculosis* through a transcriptional repressor. This enzyme is important for the integrity of the bacterial cell wall [20].

Anti-TB drugs are generally well tolerated; however, toxic side effects may occur in up to 80% of patients with tuberculosis. Hepatotoxicity linked to isoniazid, rifampicin, and pyrazinamide is the most prevalent adverse effect of anti-TB medications [21]. Multiple drug regimens enhance the adverse effects associated with anti-TB therapy. Consequently, while isoniazid, rifampicin, and pyrazinamide are individually capable of causing hepatotoxicity, their combined administration amplifies this toxic effect. Adverse reactions to anti-TB medications are linked to factors including dosage, timing of administration, patient genotype, age, nutritional status, and the presence of pre-existing conditions such as alcohol use disorder, impaired renal and hepatic function, and HIV co-infection [22,23].

### 2.2. Second-Line and Newer Drugs for Drug-Resistant TB

Over the past few decades, there has been a growing concern about the development of drug-resistant TB due to a number of factors, such as the widespread inappropriate or ineffective use of antimicrobials to treat TB in the absence of drug-susceptibility testing, the inadequate adoption of systematic approaches for treatment of both drug-susceptible and drug-resistant TB, the introduction of HIV into areas where drug-resistant TB already existed, provider error, poor treatment adherence, a shortage of effective medications, and the spread of DR strains [24,25]. According to the most recent data, just 56% of the 156,000 people with MDR-TB or rifampicin-resistant TB who started treatment worldwide in 2018 successfully finished it. The prolonged use of second-line anti-TB medications, which are less effective and more toxic than first-line agents, might be one cause of these low treatment completion rates [24]. The literature indicates that the length of therapy, the variety of medications, and polytherapy are all possible obstacles to treatment adherence, and that shortening the period of treatment may aid in enhancing treatment adherence [26,27]. According to earlier research, noncompliance with treatment can raise the chance of recurrence and the development of drug-resistant TB [28]. According to a Ghanaian study, the primary obstacles to TB treatment adherence point to significant implementation gaps in the TB program, including those pertaining to social support, food security, income stability, knowledge, and accessibility to treatment facilities. The authors concluded that the government and the National Tuberculosis Programme must work with other sectors to offer TB patients comprehensive health education, social and financial assistance, and food help in order to increase treatment adherence [29].

Drug-resistant TB, including MDR-TB and pre-XDR-TB, is now primarily treated with a six-month regimen of bedaquiline, pretomanid, linezolid, and moxifloxacin, provided the patient has not been heavily pre-exposed to these drugs. In special cases—such as fluoroquinolone resistance, pregnancy, or in children—an alternative six-month regimen may include delamanid, levofloxacin, and clofazimine. For fluoroquinolone-susceptible cases, several nine-month options exist, with combinations based on bedaquiline, linezolid, a fluoroquinolone, pyrazinamide, and occasionally delamanid or clofazimine. If newer regimens are not suitable, a standard nine-month treatment or longer individualised regimens (≥18 months) using Group A (bedaquiline, linezolid, fluoroquinolones), B (clofazimine, cycloserine), and C drugs may be needed. Emerging alternatives, such as host-directed therapies and therapeutic vaccines, are under investigation to improve outcomes and reduce treatment duration in drug-resistant TB [16].

### 2.3. Prophylaxis and Pharmacological Considerations in Special Populations

In order to prevent active disease, persons with a suspected *Mycobacterium tuberculosis* infection are usually administered preventive medication, which remains valuable to the eradication of TB. Even while the length of TB prevention treatment has been decreased over time, it is still lengthy in absolute terms, and its uptake is still low. Effective regimens lasting one to four months have been implemented thus far as a result of treatment-shortening trials employing non-inferiority designs [30]. According to WHO guidelines, preventive therapy for TB is recommended only in those cases in which the person has been in contact with a TB patient but does not have active TB. Thus, before initiation, clinical examinations and diagnostic tests are required, as appropriate. The main indications are persons with HIV, persons in direct contact with a TB patient, immunosuppressed patients, or persons in vulnerable groups [16]. Although new regimens are a significant improvement over the old standard of care, which lasted 6 to 9 months, they are still far too long considering the possibility of toxicity and the modest baseline risk of disease for the majority of people. Even while the effectiveness of further shortened tuberculosis preventive medication regimens—including ultra-short regimens lasting less than two weeks—has not yet been investigated, optimal results for public health may still be attained even if these regimens’ efficacy is inferior to that of the standard of care [30,31].

For patients aged over 12 with drug-susceptible TB, a 4-month regimen of isoniazid, rifapentine, moxifloxacin, and pyrazinamide is recommended: 2 months with all four drugs, followed by 2 months without pyrazinamide. In children aged 3 months to 16 years with non-severe, drug-susceptible TB, a 4-month regimen is also advised: 2 months of isoniazid, rifampicin, and pyrazinamide (±ethambutol), then 2 months of isoniazid and rifampicin [16].

HIV/AIDS patients have a much higher risk of having active TB. However, because many countries lack monitoring data, it is challenging to determine the incidence of HIV/TB coinfection [32]. HIV-positive patients should follow the same TB treatment duration as HIV-negative individuals. The standard six-month regimen is preferred, especially for those with CD4 <100 cells/mm^3^. A four-month regimen may be used in selected patients with CD4 >100 cells/mm^3^. Antiretroviral therapy should begin within two weeks of starting TB treatment, regardless of CD4 count [16]. Frequent testing for both latent and active TB infection is necessary due to the elevated risk of TB development in people with HIV. A multimodal strategy is needed to manage concurrent TB and HIV infections, which includes limiting immune reconstitution inflammatory syndrome, treating any coexisting medical disorders, and using anti-TB drugs in addition to antiretroviral drugs [33]. Clinicians must take into account the possible cytotoxicity and drug–drug interactions among the different therapies due to the complex design of this treatment plan [34]. Additionally, the length of treatment is a significant factor, with shorter regimens preferred when possible to improve patient compliance. The significance of personalised and comprehensive care for individuals co-infected with TB and HIV is underscored by the fact that the selection of preventive TB therapy for individuals with HIV is dependent on their individual antiretroviral regimen [33,35].

## 3. Nutritional Status and Tuberculosis

Regarding the body’s reaction to the pathogenic organism, nutrition plays a significant role in the treatment of ailments that are both acute and chronic. Numerous nutrients, including vitamins, macro- and micronutrients, and others, are linked to strengthening the host’s defences against intracellular infections, such as *Mycobacterium tuberculosis*. Any type of nutritional deficiency can result in nutritionally developed immune deficiency syndrome, which significantly raises a person’s vulnerability to infectious diseases. These nutrients also have immunomodulatory effects in regulating both the inflammatory and infectious processes [36,37]. In this direction, malnutrition has been shown in numerous studies to have negative impacts, increased vulnerability to infections, slowed recovery, and an increased likelihood of comorbidities [38,39]. Alterations in eating habits, digestion, and absorption are linked to malnutrition, which is linked to worse prognoses. Furthermore, the ability to manage oxidative stress triggered by insufficient health conditions and drug–nutrient interactions can also be strongly impacted by nutritional status [40]. Conversely, several diets have been recognised to interact with frequently prescribed pharmacological agents, presenting an additional perspective that may result in significant nutritional status alterations [41,42,43].

**Figure 1:**
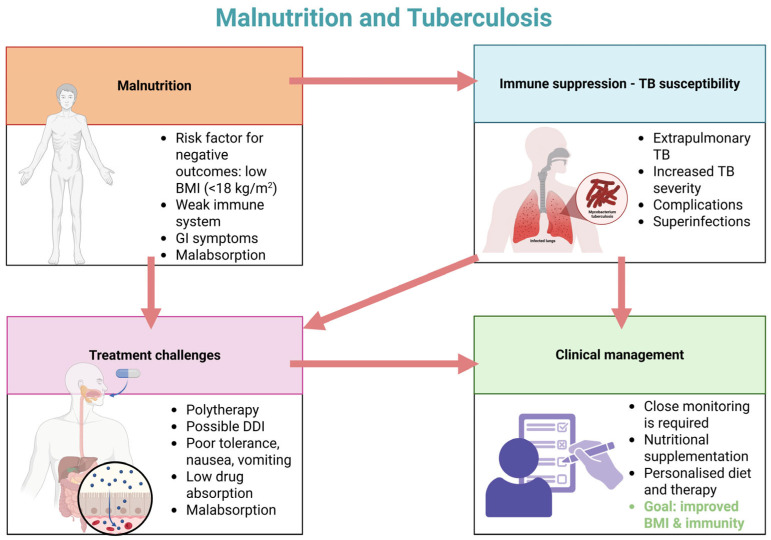
Vicious cycle of malnutrition and tuberculosis: immunity, treatment barriers, and management strategies (adapted from [46]) (created with BioRender.com).

Although malnutrition is commonly thought of as a lack or excess of a specific nutrients, in the context of global health research, it implies more broadly to a variety of life-threatening diseases in which the body is weakened or under stress due to the feedback loops that exist between inadequate nutrient intake, the immune system, and broader physiologic processes [44]. Malnutrition and tuberculosis have a complex relationship because each illness can exacerbate the other, leading to an unending chain of detrimental effects on one’s health, both of which are two interconnected global health issues that have considerable effects on public health [45] (Figure 1).

### 3.1. Malnutrition, TB Risk, and Impact on Pharmacotherapy

Generally, microbial agents cause an inflammatory response throughout the infectious cycle, which leads to fever, decreased appetite, elevated catabolism, and abnormalities in intestinal absorption. In addition to increasing nutritional requirements, these changes may trigger or worsen malnutrition. In turn, malnutrition weakens the gut barrier and further raises the risk of infection [47,48]. This alters the gut flora, impairs immune cell activation and production, changes the control of inflammatory adipocytokines, and restricts the absorption of macro- and micronutrients. By reducing lipid tissue, undernutrition affects the generation of adipokines and causes impairments in both innate and adaptive immunity. Leptin production was downregulated, and adiponectin production was increased in starving conditions. Adiponectin reduces T cell responses and B cell generation by increasing secondary macrophage activity and secreting anti-inflammatory cytokines [49,50]. Moreover, malnutrition-induced downregulated leptin and increased adiponectin synthesis, along with compromised stress hormone production, limit immune cells’ pro-inflammatory responses [51]. In cases of protein-energy malnutrition, humoral and cell-mediated immunity are significantly weakened, but with an appropriate diet, they are able to be restored. Malnutrition can impact immune system development even before conception, which is becoming increasingly acknowledged as it was demonstrated that maternal hunger produces epigenetic changes in children [38]. In this direction, researchers sought to determine how food consumption could influence epigenetic alterations that ultimately affect human health. A potential explanation involves directly influencing the catalytic activity of the enzymes that mediate the writing or erasing of epigenetic changes. Another mechanism refers to metabolically produced intermediates that can interfere with histone residues typically changed by conventional epigenetic marks [52].

Nutrient intake has a significant impact on T-cells, and modifications in the general nutritional status, such as obesity or malnutrition, can affect T-cell metabolism and behaviour. T-cell utilisation of glucose and metabolism, as well as T-cell survival, division, and proinflammatory cytokine production, are all reduced in conditions of acute malnutrition or starvation. Malnutrition-induced changes in T-cell activity and metabolism are linked to changes in adipokine levels, most notably a drop in leptin, which has been demonstrated to be an invaluable intermediary between immunity and nutrition [53,54,55]. It is commonly known that protein-energy deficiency is linked to several immune response abnormalities, such as T-cell count, T-cell subset proportion, NK cell activity, and cytokine generation. It has been demonstrated that malnutrition reduces T-cell activity, cytokine synthesis, and lymphocytes’ capacity to react appropriately to cytokines of any kind [56]. In particular, iron deficiency—along with long-term deficiencies in zinc, vitamin A, and vitamin D—may impair immune activation by promoting a Th2-dominant response, altering macrophage and dendritic cell maturation, and increasing IgE antibody production. On the other hand, immunological resilience, regulatory cell promotion, and tolerance induction are established when these micronutrients are sufficient [57]. During infections or chronic inflammation, the body may restrict access to certain micronutrients as part of its natural defence, a phenomenon known as nutritional immunity [58]. To eliminate harmful microbes, the immune system primarily employs two pathways: phagocytosis and the complement cascade [59]. The complement system can be activated independently to lyse pathogens, or it can tag microbes for destruction, enhancing their uptake by phagocytes through complement receptors. Both processes are impaired in malnutrition, leading to a decrease in opsonic complement factor C3 and a markedly diminished capacity for phagocytic ingestion and pathogen killing. In addition, malnutrition also reduces the function of macrophages, B lymphocytes, dendritic cells, and Kupffer cells, all of which deliver antigens [36,60,61]. Additionally, protein-energy malnutrition is a systemic disorder that results in the atrophy of lymphoid tissues. The thymus is one of the organs most affected, with a significant loss of thymocytes, especially CD4+ CD8+ cells. Histological examination revealed that the lymphoid and microenvironmental regions endure degeneration, which results in thymic atrophy. The thymic intralobular extracellular matrix, which contains fibronectin, laminin, and type IV collagen, was found to rise in children who were extremely malnourished; however, no cause-and-effect relationship between the increase of this extracellular matrix and the rate of thymocyte depletion has been established [62]. Moreover, as a result of protein-energy malnutrition, the thymus and peripheral lymphoid organs atrophy, which causes leukopenia, a drop in the CD4/CD8 ratio, an increase in CD4 and CD8 double-negative T cells, and an increase in immature T cells in the peripheral circulation. It was also shown that the expression of CD25 and CD27, molecules necessary for T cell activation and proliferation, had significantly decreased under these conditions [62,63]. Additionally, severe malnutrition is associated with a reduction in delayed-type hypersensitivity reactions, reflecting impaired T-cell–mediated immunity, as evidenced by diminished responses to tuberculin skin testing. In contrast, individuals with moderate malnutrition often maintain relatively intact humoral responses, including the ability to generate antibodies following immunization. Malnutrition also skews cytokine production toward a Th2-dominant profile. Despite these impairments, total leukocyte and lymphocyte counts often remain unchanged, and immunoglobulin levels—particularly IgA—are frequently elevated, suggesting that certain components of the immune system may remain intact or even compensatory enhanced [64].

The historical basis for immune support through micronutrients stems from vitamin C deficiency and supplementation in scurvy throughout earlier periods. It has now been determined that the integrated immune system requires several specific micronutrients, such as zinc, iron, copper, selenium, folate, vitamins A, D, C, E, B6, and B12, which all play significant, frequently complementary functions at every stage of the immunological response. The daily micronutrient intakes required to sustain immune function may exceed the currently suggested dietary requirements. However, adequate levels are necessary to ensure the normal function of immune cells and physical barriers. Certain populations cannot obtain enough micronutrients from their diet, and conditions that cause higher needs (including sickness, anxiety, stress, and pollution) further deplete the body’s storage. Immunity may be weakened by even slight deficiencies in micronutrients [65]. Through the action of nuclear retinoic acid receptors, all-trans retinoic acid, 9-cis retinoic acid, or other metabolites, vitamin A plays an important part in the control of innate and cell-mediated immunity as well as antibody reactivity [66]. This function is demonstrated when there is a vitamin A deficiency and an increased risk of infection [67]. The synthesis of antibodies and the proper release of cytokines are both impacted by vitamin A deficiency [68]. Furthermore, low levels of NK cells, monocytes, or macrophages, as well as poor T- and β-lymphocyte maturation and proliferation, are linked to vitamin A deficiency. By preventing the normal rebuilding of infection-damaged mucosal barriers and by reducing the activity of neutrophils, macrophages, and natural killer cells, vitamin A deficiency compromises innate immunity [69].

Furthermore, in addition to being a necessary micronutrient, iron is also required for producing ROS, which is a process through which neutrophils, monocytes, or NK cells employ iron as a catalyst to combat infections. In fact, complex I and III of the mitochondrial electron transfer chain, which contain metal centres mainly in the form of iron-sulphur clusters, or other intracellular sources like peroxisomes and the endoplasmic reticulum, or iron-loaded lactoferrin that releases ferrous iron, are the three main sources of ROS during infections. Thus, ROS is produced on demand, and iron shortage prevents these enzymes from functioning properly. Even though these cells exhibit enhanced activity, microbicidal death is impeded in iron-deficient settings because ROS generation is inhibited. IL6, TNFα, and IFN-γ are examples of Th1-signatures that are first linked to iron deficiency. Th2 skewing and a rise in the cytokine IL4 are linked to a persistent deficiency, as observed in severe forms of iron-deficient anaemia [58,70,71]. During oxidative stress, such as that experienced in phagocytic cells, iron can be released from intracellular proteins and catalyse the Fenton reaction, generating highly reactive oxygen species. While this mechanism enhances microbial killing, some pathogens may exploit the transient increase in intracellular free iron to support their own growth [72]. Additionally, a zinc deficiency affects Th cell polarisation by encouraging the differentiation of Th0 cells into proinflammatory Th17 cells, which results in a loss of Treg function and thymic atrophy, decreased lymphocyte numbers and activity, altered synthesis of cytokines, elevated oxidative stress, and inflammation [73,74]. In the same direction, reduced neutrophil numbers and antimicrobial activity, as well as decreased T cell IL-2 release and subsequent T cell proliferation, are linked to copper deficiency [75].

A recent meta-analysis concluded that undernutrition likely doubles the risk of TB in the short term (less than 10 years) and may also raise the incidence in the long term (more than 10 years). Not only are policies necessary to alleviate the distress of humans as a result of undernutrition and its numerous adverse effects, but they are also an integral part of the efforts necessary to end the worldwide epidemic of TB by 2030 [76]. In Table 1, we summarise recent studies that investigated the association between a low body mass index (BMI) and TB incidence.

**Figure 2:**
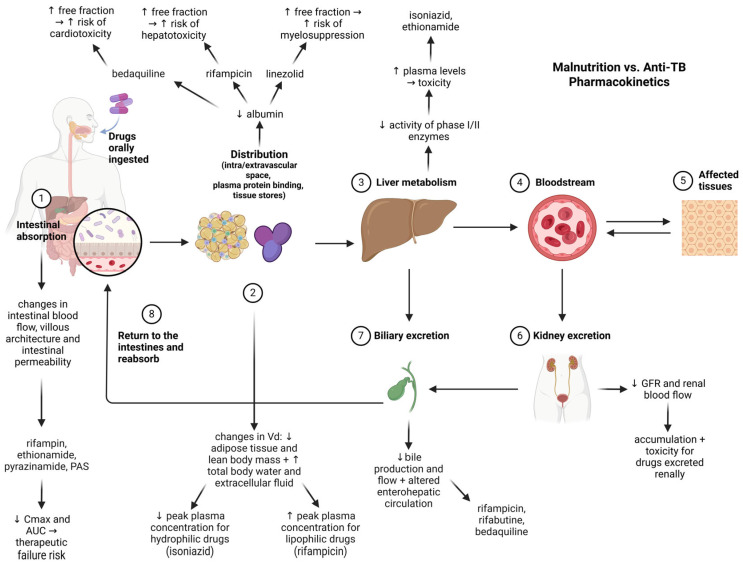
Impact of malnutrition on pharmacokinetics of antituberculosis drugs (created with Biorender.com). Legend: Cmax—maximum plasma concentration of a drug; AUC—Area Under the Curve (describes total drug exposure over time); Vd—volume of distribution (a pharmacokinetic parameter that indicates extent of drug distribution in body tissues); GFR—glomerular filtration rate (an indicator of kidney function); PAS—para-aminosalicylic acid. Solid arrows indicate direction of drug movement or physiological flow through pharmacokinetic pathway. Upward (↑) and downward (↓) arrows next to drug names or physiological terms indicate increases or decreases in parameters (e.g., absorption, plasma levels, protein binding, enzymatic activity) induced by malnutrition.

Malnutrition is a primary risk factor for TB and can also have detrimental effects on anti-TB drug therapy. Figure 2 illustrates the primary physiopathological alterations induced by malnutrition that can affect the pharmacokinetics of anti-TB agents.

Multiple lines of evidence suggest that malnutrition alters the absorption, distribution, metabolization, and elimination processes [83] and impacts anti-TB drug pharmacokinetics [84]. Malnutrition reduces intestinal absorption via changes in gut architecture and permeability, affecting rifampin, ethionamide, pyrazinamide, and PAS [85,86]. Altered body composition and hypoalbuminemia change drug distribution, increasing the free fraction and toxicity risk (e.g., bedaquiline, rifampicin, linezolid) [83,86,87]. Impaired liver metabolism elevates plasma levels of hepatically cleared drugs (e.g., isoniazid, ethionamide) [83,88]. Malnutrition is associated with reduced glomerular filtration rate and renal blood flow, impairing the elimination of renally-excreted drugs and increasing their systemic accumulation and toxicity [83]. Reduced bile production and flow, along with changes in enterohepatic recirculation, affect the excretion and reabsorption of drugs like rifampicin, rifabutin, and bedaquiline [83,89]. Therefore, routine assessments on nutritional status before treatment initiation and therapeutic drug monitoring should be conducted by healthcare professionals, as these can cause subtherapeutic drug levels or increased toxicity.

It is noteworthy that malnutrition is not only a result of *Mycobacterium tuberculosis* infection but also a well-established risk factor for its development. Many individuals—particularly in high-burden areas—show signs of underlying undernutrition before TB diagnosis. By weakening epithelial barriers, thus compromising immune responses, and so changing systemic metabolic pathways, this reduced nutritional status provides a biological milieu fit for infection. Moreover, as it alters medication absorption, distribution, metabolism, and excretion—variables that might result in subtherapeutic drug levels, higher toxicity, and worse treatment efficacy—malnutrition also affects the pharmacological therapy of TB (Figure 2).

### 3.2. The Effect of TB Infection on Nutritional Status and Common Nutritional Deficiencies in TB Patients

While TB patients exhibit both humoral and cell-mediated immune responses, effective control of *Mycobacterium tuberculosis* relies predominantly on cell-mediated immunity. This includes the activation of macrophages by IFN-γ-producing Th1 cells, cytotoxic T lymphocytes, and the formation of granulomas to contain the infection. Although antibodies are produced, their protective role in TB remains limited and not fully understood [90]. It has been demonstrated that B-cell deficiency increases the bacterial load and worsens the prognosis after *M. tuberculosis* infection, whereas antibodies to *M. tuberculosis* promote phagocytic cells’ internalisation of mycobacteria. These antibodies result in a boost in *M. tuberculosis*-specific cell-mediated immunity and considerably enhance macrophages’ capacity to eliminate intracellular mycobacteria [91]. The host immune response to *M. tuberculosis* infection is coordinated by a variety of inflammatory cytokines from both the adaptive and the innate immune systems. These immune responses strain the host considerably metabolically [92]. Commonly reported in TB patients, the persistent inflammation, cytokine-driven catabolism, and nutritional redistribution linked with the immunological response enable the explanation of the gradual wasting and micronutrient deficits. This immuno-nutritional interaction not only reduces clinical outcomes but also compromises immunological defences, therefore aggravating undernutrition and infection in a vicious cycle [93]. In this direction, patients with tuberculosis typically are identified with a metabolic wasting syndrome, which causes marked weight loss and an abrupt reduction in muscle mass [94]. Additionally, a low BMI is frequently observed in TB patients, and multiple lines of evidence consistently demonstrate that low BMI is strongly associated with an increased risk of mortality during anti-TB treatment, as presented in Table 2.

Changes in the host’s energy metabolism during TB infection are marked by the downregulation of enzymes involved in oxidative phosphorylation and the tricarboxylic acid cycle, and the upregulation of important glycolytic enzymes and carriers for glucose uptake [103]. Shi et al. also found an overexpression of a lactate secretion transporter and a V-type H+-ATPase associated with cytosolic pH homeostasis, consistent with increased glycolysis. Transcriptional profiling revealed elevated expression of glycolytic enzymes and increased HIF-1α levels, both at the mRNA and protein level, in macrophages and T cells within granulomatous lesions. Accordingly, these results imply that immune cells respond to *M. tuberculosis* infection mostly by using aerobic glycolysis [104]. For TB patients, these metabolic alterations clinically manifest through an increased energy demand and ineffective energy generation, which promotes malnutrition and muscle loss. Reducing ATP yield per glucose molecule and increasing lactate generation from oxidative phosphorylation to aerobic glycolysis (the Warburg effect) aggravates the catabolic condition. Basically, this means that in order to maintain tissue repair and immunological function and to counterbalance the metabolic shift, TB patients may need to consume more protein and calories [105]. It is unclear if, following infection-induced immunological tolerance, the TCA cycle also controls epigenetics, particularly DNA methylation. The TCA cycle mediates the DNA methylation alterations caused by lipopolysaccharide and *M. tuberculosis*. Inhibitors of the TCA cycle and cellular metabolism reduced infection-induced DNA hypermethylation. On the other hand, immunological tolerance and DNA hypermethylation were induced by exogenous intake with TCA metabolites, succinate, and itaconate [106]. Different TCA cycle activity and accumulation of particular metabolites could affect immunological tolerance and epigenetic control, therefore influencing immune dysfunction or ongoing inflammation. This implies that an imbalance of some micronutrients and metabolic byproducts may trigger epigenetic alterations and immunological suppression [107].

At a tertiary care hospital in India, Mamadapur et al. conducted a two-year prospective case–control study with 116 participants: 58 newly diagnosed TB patients (29 pulmonary, 29 extrapulmonary) and 58 healthy controls. Vitamin D levels were compared between cases and controls using statistical analysis and standardised laboratory testing, with participants matched by age and sex. According to the study, vitamin D deficiency was more common in TB patients than in non-TB patients [108]. In the same direction, according to a study by Pareek et al., vitamin D insufficiency was linked independently to an increased risk of extrapulmonary dissemination, and those with extrapulmonary TB had considerably lower blood 25(OH)D levels than people with pulmonary TB. Extrapulmonary TB was found to be independently linked to vitamin D deficiency, indicating that the immune system’s ability to control *Mycobacterium tuberculosis* at the original pulmonary location was compromised, allowing for haematogenous spread [109]. Cathelicidin, an antimicrobial protein, exhibits antimicrobial action against bacteria and viruses, including *M. tuberculosis*. Mechanistically, it has been proposed that the induction of cathelicidin is necessary for the vitamin D-mediated antimicrobial effect against *M. tuberculosis*. Vitamin D additionally increases the production of cathelicidin in a variety of cell types, including macrophages [110]. Autophagy is activated by the vitamin D–cathelicidin axis, which strengthens antimicrobial activity against a variety of pathogens. Physiologically, autophagy is activated by vitamin D/vitamin D receptor signalling via a variety of mechanisms, such as suppression of the mTOR, a negative regulator of autophagy, and intracellular calcium release/calcium-dependent kinases. Interestingly, the AMPK pathway mediates autophagy connected to functional vitamin D receptor signalling through the lipoprotein LpqH, a mycobacterial TLR2/1 agonist [111]. Vitamin D3 conditioning reduces host cytotoxicity, increases bacterial clearance during low-level infection, and increases intracellular containment during high-level infection, according to an in vitro study by Gough et al. The production of cytokines and effector molecules in the presence of vitamin D3 depended on the infection level. Notably, NO had a rate of change that was positively linked with IL-12, and IL-12 rose during high-level infection and reduced during low-level infection. Thus, the study shows that vitamin D3 regulation is time- and context-dependent and strongly associated with infection level [112]. Therefore, it has been suggested that vitamin D deficiency facilitates increased bacterial burden, which in turn alters the host immune response.

In CD4⁺, CD8⁺, and NK cell populations, DiNardo et al. showed that *Mycobacterium tuberculosis* causes ongoing DNA hypermethylation of immune signalling pathways including IL-12/IFN-γ, TNF/NF-κB, and IL-2/STAT5. Following both mitogen and antigen stimulation, this epigenetic remodelling significantly reduces the synthesis of antituberculotic cytokines, including IFN-γ, TNF, IL-6, IL-1β, CXCL9, and CXCL10. Hypomethylated genes, including IL12B, IL12RB2, TYK2, IFNGR1, JAK1, and STAT4, were linked to reduced cytokine inducibility and immune cell proliferation. These alterations suggested long-lasting immunological exhaustion as they continued even six months after TB treatment was finished [113]. Additionally, *M. tuberculosis* secretes a functioning DNA methyltransferase, Rv2966c, which penetrates host immune cells and directly causes hypermethylation of host immunity genes, hence contributing to this immunosuppressive condition [114]. These processes offer a reasonable theory for the higher bacterial burden seen in TB patients as well as their suppressive effect on protective cytokine responses.

On the other hand, Kumar et al. demonstrated that elevated proinflammatory cytokine levels (IFN-γ, TNF-α, IL-17A, and IL-1β) are characteristic of TB and that anti-TB therapy can modify these indicators, which are characteristic of the disease severity and the bacterial load. Since elevated levels of both IFNγ and TNFα are seen in TB patients, elevated type 1 cytokine levels might be a characteristic of disease severity. A significant correlation between IFNγ and TNFα and the severity of TB disease, as well as the degree of pathology, was established. Lastly, a negative connection with time to culture conversion and a direct correlation between IFNγ and TNFα levels and bacterial burdens, indicating that type 1 cytokines in TB are primarily influenced by infection density [115]. TNFα, IL-1, IL-2, IL-6, IL-8, and IFNγ are among the pro-inflammatory cytokines that reduce appetite. Anorexia and hypermetabolism driven by cytokine-induced appetite suppression are significant causes of chronic illness cachexia and a component of the body’s acute phase reaction to different immunological stimuli. During an immunological assault, acute, transient anorexia is thought to be advantageous for the host. However, persistent cytokine-mediated anorexia might impair the host’s capacity to combat infection [116]. In a study conducted by Pourhassan et al., 30% of the subjects had a CRP of greater than 3.0 mg/dL, 31% were malnourished, and 31% had low or very poor appetite. The mean concentrations of several cytokines, including as IL-1β, MCP-1, IL-6, IL-10, IL-12p70, IL-18, and IL-23, varied significantly throughout appetite scores. The most significant biomarker for low appetite was an elevated IL-18 level [117]. Mechanistically, proinflammatory cytokines affect the metabolism of muscle proteins by downregulating anabolic pathways and stimulating catabolic ones. For instance, elevated blood levels of TNF-α and IL-1 are linked to lipopolysaccharide-induced muscle atrophy, which inhibits the Akt/mTOR. Proinflammatory cytokines interact with the IGF-1-dependent signalling pathway to partially exert their antianabolic effects. TNF-α triggers IRS-1 to become serine phosphorylated, which prevents it from binding to the insulin/IGF-1 receptor. Through direct contact between IRS-1 and IKK complexes, TNF-α can affect insulin/IGF-1 signalling [118].

Table 3 provides an overview of the most frequently reported nutritional deficiencies in patients with TB, highlighting the impact of the disease on micronutrient and macronutrient status.

## 4. Deficiencies Induced by Anti-TB Drugs and Drug-Nutrient Interactions

### 4.1. Drug-Induced Nutritional Deficiencies

Drug-induced nutritional deficiencies represent a significant yet underappreciated class of adverse drug reactions, which are any unintended consequences of using a medication that increases toxicity, reduces therapeutic efficacy, or both. The majority of reports highlight adverse drug reactions that are caused by errors in dosage frequency, interactions between drugs, and administration or dispensing procedures. Therefore, such consequences should be weighed against the expected benefit of every therapy choice. The majority of adverse drug reactions have mechanism-based or idiosyncratic aetiologies [137,138].

The micronutrient status of a patient may be directly or indirectly impacted by medications. Because they may utilise the same metabolic and transport routes in the body, certain drugs may have a direct impact on the pharmacokinetic characteristics (absorption, distribution, metabolism, or excretion) of micronutrients. Additionally, the micronutrient may be directly impacted by physiologic alterations caused by the drug’s mode of action. Because of its impact on the patient’s general nutritional state (weight gain, weight loss), metabolic status (hyperglycaemia, hypertriglyceridemia), or specific micronutrient or mineral status (hypokalaemia, zinc deficiency), the drug itself may have an indirect impact on the patient’s health [139]. The metabolism of nutrients, including their absorption, distribution, catabolism, excretion, and conversion to active forms, can be impacted by various pharmacological agents. There are a lot of potential tissue-specific interactions since different tissues have compound-specific transport proteins, receptors, and enzymes. This makes it challenging to predict clinical consequences because it changes the pattern and location of where nutrients and medications interact [140] (Table 4).

Without confirming nutrient deficiencies or determining that a patient is at significant risk for nutrient deficiencies, widespread nutrient supplementation is not advised. It should be determined if a nutrient shortage is preclinical or clinically relevant before assessing any drug-induced deficiencies. In the event that this shortfall is not remedied, it should be assessed whether there might be any potential health implications or if the deficiency is very mild and unlikely to result in any health consequences. Moreover, there are no established protocols for managing and/or preventing nutritional deficiencies induced by drugs. It is imperative to guarantee that at-risk patients obtain the necessary quantities of essential vitamins and minerals, despite the fact that general supplementation is not the current recommendation [140,155].

### 4.2. Drug–Food Interactions in Tuberculosis Therapy

A drug’s physical, chemical, physiological, or pathophysiologic associations with a food component are referred to as drug–food interactions. Most clinically important drug–food interactions have several underlying causes. Negative effects on patient outcomes and extremely significant ramifications might result from improperly identifying and managing drug–food interactions [156] (Table 5).

Food–drug interactions may unintentionally decrease or amplify the effects of medications. Certain frequently used fruits, vegetables, herbs, and alcohol can lead to therapeutic failure and even major changes in the patient’s health. Most clinically significant food–drug interactions originate from dietary modifications to the drug’s bioavailability. Changes in absorption caused by fatty, high-protein, and high-fibre diets are among the significant adverse effects of certain foods on medications. The therapeutic effect of the majority of drugs relates to bioavailability. The most noteworthy are the interactions linked to an elevated probability of treatment failure due to a markedly decreased bioavailability in the fed state. Chelation with food components is often the cause of such interactions. Furthermore, the physiological reaction to meal consumption, specifically the production of stomach acid, may decrease or enhance the bioavailability of some medications [162,166,167].

Drug–food interactions should be routinely identified as a component of the patient evaluation or the drug regimen review in order to maximise a patient’s clinical outcomes. This calls for a general understanding of the drug–food interactions framework that goes beyond a few specific instances. The healthcare professional might become more proactive in predicting possible interactions by expanding their knowledge of the potential mechanisms of interaction. Certain interactions (such as tyramine-containing meals with inhibitors of monoamine oxidase or calcium-containing food products with ciprofloxacin) may be seen as separate facts by certain clinicians, who may fail to see how each fits into the broader classification scheme [168]. Time restraints, shorter hospital stays, and a lack of knowledge about the numerous medication formulations and interactions are some of the factors that limit comprehension and provider efforts. To help patients avoid drug–food interactions, nutritionists, pharmacists, and doctors must use interdisciplinary efforts and a coordinated strategy. It is anticipated that healthcare practitioners would advise patients on how to take their medications in connection with food, how to take them, and when to take them in relation to meals [169].

## 5. Nutritional Management of Tuberculosis Patients

As detailed in Section 3, poor nutrition exacerbates TB outcomes, and TB further exacerbates nutritional status, a vicious cycle that is particularly prevalent in low-resource environments.

Increased immune responses against intracellular pathogens such as *M. tuberculosis* are linked to a variety of nutrients. In order to regulate the process of infection and inflammation, these vitamins, minerals and trace elements have an immunomodulatory effect. Lack of protein-energy, or micronutrients, alters immunological homeostasis, making a person far more vulnerable to infections or the progression of infectious conditions into diseases [36]. According to WHO, material support for malnutrition or undernutrition should be regarded as equally imperative as other TB treatments when treating TB patients. Moreover, the aetiology of undernutrition must be evaluated, and socioeconomic issues must be addressed if economic hardship or food insecurity are the main contributing factors. A suitable medical referral ought to be taken into consideration when underlying medical causes are detected [16]. No evidence suggests that the dietary management of acute malnutrition in patients with active tuberculosis should differ from that of patients without active tuberculosis. Concerns regarding weight loss or inadequate weight gain necessitate additional clinical evaluation (e.g., resistance to TB medications, poor adherence, comorbidities) and nutritional assessment to identify the most suitable interventions. Enhanced nutritional monitoring and the earlier introduction of nutritional support, prior to the completion of the initial two months of TB treatment, ought to be provided when the nutritional indicator nears the threshold for diagnosing severe undernutrition [170].

As noted in Table 3 and Table 4, *Mycobacterium tuberculosis* infection can lead to considerable nutritional deficits, whereas routine pharmaceutical therapies may also independently induce nutritional deficiencies. This promotes the establishment of a well-founded nutritional management plan for tuberculosis patients, considering the various factors that might influence their nutritional status. The WHO states that in order to avoid or manage isoniazid-associated neurotoxicity, pyridoxine fortification may be necessary. It is typically not necessary to routinely administer pyridoxine supplements to otherwise healthy people on a regular dosage of isoniazid. A healthy diet that includes 1–2 mg of vitamin B6 molecules per day may help prevent isoniazid toxicity. Carrots, spinach, peas, potatoes, milk, cheese, eggs, fish, meat, and fortified flour are all good sources of vitamin B6. People who are at risk of developing peripheral neuropathy can avoid it by taking pyridoxine and isoniazid at the same time; 10–25 mg per day is the suggested dosage. Pyridoxine should be administered at a greater dose of 50–75 mg daily, and even up to 100–200 mg daily, for persistent isoniazid-induced peripheral neuropathy. Maintaining pyridoxine supplementation at the proper dosage is of great importance since higher amounts may compromise isoniazid’s antibacterial action. Furthermore, pyridoxine toxicity, including peripheral neuropathy, has been linked to overdose (≥2000 mg/day) [171]. In this direction, in a prospective study involving 285 treatment-naïve tuberculosis patients in Nigeria, those who received methionine and vitamin B-complex alongside standard antitubercular therapy showed significantly better clinical and biochemical outcomes compared to the control group (standard therapy alone). After 6 months, the test group had higher red blood cells and packed cell volume, and at 2 months, they exhibited lower liver and kidney function markers (AST, ALT, ALP, urea, creatinine, and total bilirubin). Additionally, antioxidant levels (reduced glutathione and superoxide dismutase) were significantly higher, and the oxidative stress marker malondialdehyde was lower. The test group experienced fewer adverse drug reactions and no hepatotoxicity, compared to the control group. These findings suggest that methionine and vitamin B-complex supplementation can mitigate toxicity, enhance antioxidant defences, and improve treatment tolerability in TB patients [172].

Some studies recommend 35 kcal/kg bodyweight per day for TB patients who are susceptible or diagnosed with malnutrition, although the available research does not identify appropriate calorie and protein objectives. The ESPEN recommends 30 kcal/kg bodyweight and a minimum of 1 g protein/kg bodyweight per day, with changes according to disease-specific or age-related corrections, personalised screening, and monitoring. Medical nutrition should provide micronutrients at the recommended daily intakes. Only patients with confirmed deficits should get sustained micronutrient supplementation. Nutritional support during TB treatment should be encouraged to increase treatment adherence and be part of the normal management of TB and MDR-TB. Nutritional rehabilitation may prevent TB-associated comorbidities and increase health outcomes [173]. Patients ought to receive promptly provided dietary support, counselling, anthropometric evaluations and laboratory tests at the initial phase of treatment. Diagnosing and treating anaemia requires careful consideration, especially when it comes to iron consumption. Additionally, providing nutritional support for household contacts—food or financial transfers—should be a priority. When weight increase during treatment is insufficient, investigations should be started. After therapy ends, nutritional status should be reviewed; undernourished individuals should be under strict observation for the likelihood of relapse [174]. Bhargava et al. included patients over 18 years old with microbiologically proven pulmonary TB. Patients were prescribed micronutrient tablets and a meal plan (1200 kcal, 52 g protein per day). Drug-susceptible TB patients received nutritional care for 6 months and MDR-TB patients for 12 months. Provided their BMI was below 18.5 kg/m^2^, they were eligible for an extension of 6 months. Highly severe undernutrition cohorts received nutritional assistance. Weight increase, especially in the initial 2 months, significantly reduced TB mortality. To enhance therapeutic results in patient-centred care, nutritional support is mandatory [175]. Dietary interventions based on increased calorie and protein consumption mostly enhanced body weight, handgrip strength, and TB recovery, with some trials showing beneficial changes in body composition, according to a systematic review that included 19 studies. Various interventions were used, including food or oral supplements and occasionally counselling. Results from micronutrient supplementation were mixed, with zinc, vitamin A, and vitamin D showing no discernible advantages. Some data indicated limited benefits (vitamin D and C) or possible damage (vitamin E in smokers). Arginine and iron failed to be effective. A diet high in cholesterol seemed to positively influence sputum culture sterilisation, bacillary population, and sputum production, though it did not affect respiratory symptoms [173]. A pilot study was conducted in India with 282 active TB patients, and the results were compared to those of a control site. The intervention included monthly nutritious food baskets, dietary counselling, treatment adherence promotion, and community mobilisation. The mean weight of TB patients improved considerably throughout each meal delivery event. The average weight of the TB patients started the intervention at 42.9 kg and reached 49.3 kg by the end. Treatment success was 95% in the targeted group and 83.5% in the control group. The study highlighted that nutritional supplements and support may assist TB patients in gaining weight and improving treatment outcomes [176]. In a prospective observational study carried out in Bangladesh, the hospital provided lunch and dinner with rice, vegetables, meat or fish, and lentil soup. In the intensive phase of therapy, hospital patients also ate an egg, 250 mL of milk or one banana, and toast for breakfast. In addition to free food, diagnosis, follow-up testing, and therapy, patients received cash support each month to buy additional food of their choosing for four months. Nutritional assistance improved therapy response, and adequate nutritional support alongside therapy is expected to improve community results [177]. Shah et al. conducted a retrospective study in India, in which the quantitative component was a cohort of 645 drug-susceptible TB patients who received nutritional supplements and 645 who did not. Compared to non-supplemented patients, nutritional supplements increased cure rates and decreased mortality. The nutritional supplement group gained more weight over six months. Qualitative data showed that nutritional supplementation improved hunger, energy, and symptom resolution, while control group members suffered budgetary restrictions and diminished appetite. In conclusion, dietary supplementation improved TB treatment results, including recovery rates, mortality, and increased weight [178].

Another significant problem that claims many lives each year is drug resistance in TB patients, which significantly slows recovery. An adequate diet reduces microbial resistance through modifications to the immune system, according to research [179]. People with MDR-TB have a high rate of unsuccessful TB therapy. It is less common in people with MDR-TB who have received prior TB treatment, but it is more common as people age and when they are malnourished. Therefore, older, underweight, and recently diagnosed patients with MDR-TB may benefit from measures aimed at improving treatment results. For instance, to maximise the effectiveness of TB treatment, malnourished individuals with MDR-TB may require strict nutritional evaluation and counselling, including nutritional support [180]. In this direction, from January 2018 to December 2020, 360 patient files from four TB clinics in South Africa and one referral hospital were reviewed. Most patients had HIV and DR-TB, with rifampicin-resistant TB and MDR-TB being the most common. Treatment results varied significantly with BMI values. Malnutrition negatively affected the therapeutic outcome, with underweight patients having among the lowest cure rates (23.2%). Normal BMI patients had 34.7% cure rates, whereas overweight and obese patients showed modest outcomes. HIV co-infection further lowered cure rates, with co-infected patients performing worse. The study highlighted that dietary status strongly affects DR-TB treatment outcomes, especially in HIV-positive patients. Underweight patients have the highest risk of negative outcomes, making nutritional support indispensable for DR-TB therapy. The authors concluded that addressing treatment success discrepancies requires integrated HIV care and gender-specific strategies, while targeted initiatives could improve results in high-burden, resource-limited environments [181]. As TB and MDR-TB patients require an evidence-based nutritional intervention, multiple clinical studies are currently employed in order to address these issues. In Table 6, some recent clinical trials that assessed different interventions for the management of TB patients are presented.

A recent study conducted by Campos-Pardos et al. supports the necessity for vitamin and mineral supplementation, grounded in solid empirical data and robust guidelines and raises the concern of the impact of improper vitamin supplementation on the infection severity. In the experimental mouse model, mice on a vitamin B12-deficient diet exhibited improved survival rates and reduced bacterial presence in their lungs; however, upon administration of B12 supplements, they experienced a significant relapse of infection. *Mycobacterium tuberculosis* has lost the ability to synthesise vitamin B12 due to the evolutionary decay of its B12 biosynthetic genes, in contrast to its ancestor *M. canettii* and environmental mycobacteria. Despite this, *Mycobacterium tuberculosis* retains B12-dependent metabolic pathways, particularly those involved in methionine synthesis, and relies on host-derived B12 for full virulence [186].

**Figure 3:**
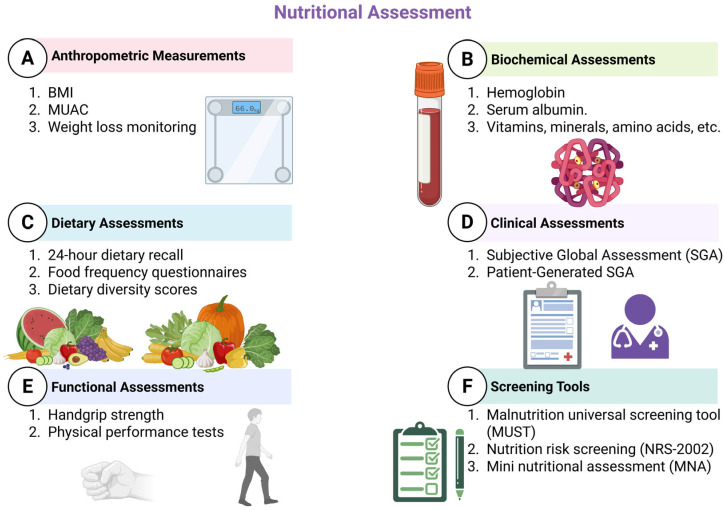
Nutritional assessment tools (created with Biorender.com). This figure summarises six main domains of nutritional assessment: Anthropometric Measurements including body mass index (BMI), Mid-Upper Arm Circumference (MUAC), and weight loss monitoring; Biochemical Assessments such as haemoglobin, serum albumin, and micronutrient (vitamins, minerals, amino acids) levels; Dietary Assessments including 24 h dietary recall, food frequency questionnaires, and dietary diversity scores; Clinical Assessments using the Subjective Global Assessment (SGA) and Patient-Generated SGA (PG-SGA); Functional Assessments like handgrip strength and physical performance tests; and Screening Tools such as the Malnutrition Universal Screening Tool (MUST), Nutrition Risk Screening (NRS-2002), and Mini Nutritional Assessment (MNA).

A nutrition-focused history and examination, an anthropometric evaluation and pertinent laboratory tests ought to be part of a nutritional assessment, according to [46]. Interpreting data from dietary and nutritional biomarkers, as well as anthropometric and clinical research, is a component of nutritional assessment systems. As impacted by the consumption and utilisation of dietary components and nutrients necessary to sustain development, restoration, and overall health, the information is employed to assess the nutritional requirements of individuals or segments of the population [187]. In the field of nutrition epidemiology, among the most important aspects is the selection of acceptable methodologies for nutritional evaluation. In the past, these techniques have been utilised to characterise the stages of deficiency; more recently, they have been utilised to identify correlations involving maintaining good health and associated risks for chronic diseases [188]. Figure 3 provides a visual representation of the most commonly used approaches to nutritional evaluation.

To preserve and enhance patients’ nutritional status, interdisciplinary approaches should be used to establish and implement nutritional treatment regimens. It is possible that standardised dietary management, which includes methodical risk assessment and screening, will lower medical expenses. Enhancing both the quality of life and functionality, as well as shorter hospital stays, lower mortality, and less severe complications, have been associated with the timely and appropriate provision of nutritional support [189,190].

## 6. Integrating Nutritional Management with Pharmacotherapy in TB Care

The effective nutritional and pharmacotherapeutic management of TB patients emphasises the need for a holistic approach to ensure increased patient care. Thus, it is becoming increasingly evident that continuous cooperation between the physician, nutritionist and pharmacist is needed to address the entire panel of problems associated with the care of the tuberculosis patient. Thus, in view of the evidence presented above regarding the different ways in which pharmacotherapeutic regimens may influence the nutritional status of the patient, as well as the possible drug–nutrient interactions that may occur during therapy, a paradigm is emerging in which dietary management should enhance pharmacologic interventions [191].

An interdisciplinary approach to managing TB can improve patient health outcomes, adherence and the efficacy of therapy [192]. In this regard, nutritionists play a particularly important role. These specialists can contribute significantly to increasing adherence to an appropriate nutritional plan for the TB patient, which may promote faster recovery rates. Thus, the development of balanced nutritional plans is necessary to improve the health status [193]. A correct and balanced nutritional intake has been positively correlated with increased treatment compliance in TB patients and has demonstrated long-term beneficial effects [194]. Furthermore, the involvement of the clinical pharmacist for the management of possible drug–drug or nutrient–drug interactions and the optimisation of pharmacotherapeutic plans and their adaptation to the needs of the TB patient is necessary in efforts to personalise therapy and increase patient-centred care. This direction is even more significant in MDR TB patients, where poor adherence to treatment significantly affects health outcomes [195,196]. Cooperation among healthcare professionals helps to ensure that drug schedules meet a patient’s dietary requirements, therefore focusing on both pharmacological and nutritional aspects of TB treatment.

**Figure 4:**
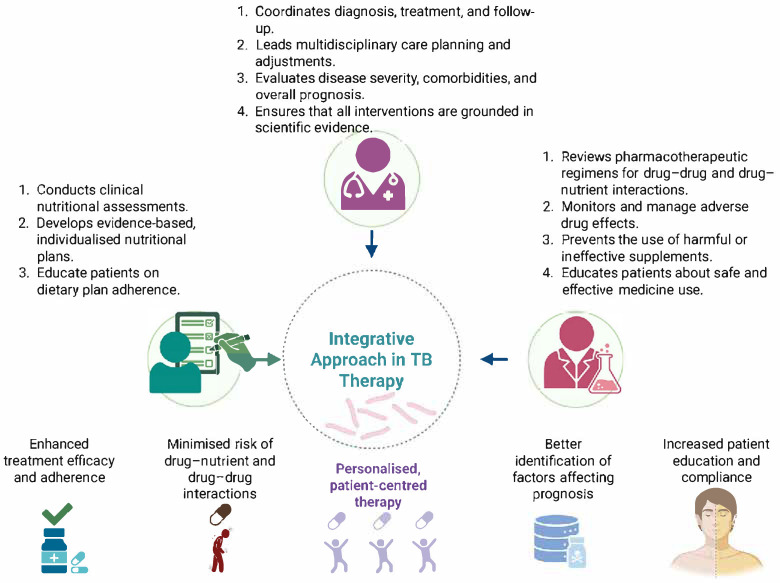
A holistic approach to ensure increased patient outcomes in TB (created with Biorender.com).

Integrating nutritional management into TB care involves clinical assessments by nutritionists, regular drug therapy reviews by pharmacists and ongoing assessments by physicians (Figure 4). In addition, healthcare plans developed within a multidisciplinary team are an important strategy in personalising therapy to meet the requirements of each patient [174]. Healthcare professionals can identify specific factors that may affect treatment adherence and overall prognosis by collaboratively developing integrated models of prediction, such as dietary restrictions, adverse drug effects, and social determinants of health [197].

In general, the emphasis on a multidisciplinary framework in TB care, which is characterised by the interconnection between pharmacotherapy and nutritional management, points to a transition to more comprehensive, patient-centred care. This approach emphasises the importance of a collaborative effort among a variety of health professionals to address the complexity of TB patients’ requirements, resulting in improved health outcomes and contributing to the global fight against TB.

## 7. Conclusions

Patients diagnosed with TB present with a complex clinical picture characterised by multiple pre-existing health problems, induced or aggravated by *Mycobacterium tuberculosis* infection. Antituberculosis treatment is associated with several significant adverse effects, which may adversely affect prognosis and therapeutic compliance. In this context, a holistic approach to the TB patient is required, integrating the expertise of the treating physician, the pharmacist and the nutritionist for a personalised and multidimensional assessment. The development of nutritional plans should be based exclusively on current scientific evidence and be based on an integrative perspective, taking into account not only individual nutritional needs, but also possible interactions between drugs and food. Correct patient information is also mandatory so that patients are educated to avoid self-medication and are encouraged to rigorously follow the recommendations of the medical team. In the documentation process for this article, we have identified a significant number of recommendations for nutritional supplements that are not supported by sound scientific data and which, in some cases, may compromise the effectiveness of treatment or even the health of the patient. In the future, more clinical research is needed in this area, multi-centre studies conducted in different countries and socio-economic settings, evaluating nutritional interventions tailored to the local context. The development of evidence-based practice guidelines sensitive to geographic variability, population characteristics and regional patterns of microbial resistance could significantly improve TB therapy for patients.

## Figures and Tables

**Table 1:** Summary of studies investigating association between low body mass index and increased tuberculosis incidence.

Study Design and Population Characteristics	BMI Categories Analysed	Outcome Measured	Confounders Adjusted For	Key Findings	Limitations	Reference
Case–control study, Central Java, Indonesia, 2016; 57 adults aged 19–63 years (19 TB cases, 38 controls); 52.6% female	<18.5 (underweight), 18.5–24.9 (normal), >25 (overweight)	TB incidence (based on AFB and chest X-ray)	Energy, protein, vitamins A/C/selenium intake, smoking, income, education, DM history	BMI <18.5: OR 6.0 (95% CI 1.32–27.18); underweight most influential risk factor	Small sample (*n* = 57), recall bias (FFQ), no biochemical validation of nutrients or albumin, limited generalizability	[77]
Retrospective cohort, South Korea, 2009–2018; 10,087,903 adults from national screening; age ≥20, 52% male	<18.5, 18.5–22.9 (ref), 23.0–24.9, 25.0–29.9, ≥30 kg/m^2^ (Asia-Pacific criteria); stratified by DM status	TB incidence (RID registration); 7.3-year follow-up	Age, sex, smoking, alcohol, physical activity, income, hypertension, dyslipidemia	Underweight: aHR 2.21 (2.14–2.28) in non-DM; aHR 3.24 (2.95–3.56) in DM; stronger effect in age <65, smokers, drinkers	BMI measured once; Korean population; possible diagnostic miscoding; no latent TB or clinical progression data	[78]
Cross-sectional study, India, 2023; 253 PTB (127 LBMI, 126 NBMI) and 176 LTB (71 LBMI, 105 NBMI); age 18–65; HIV and DM excluded	<18.5 (LBMI), 18.5–24.9 (NBMI)	Immune markers in TB and LTB	HIV, DM excluded; no adjustment for others	LBMI associated with ↓ IFNγ, IL-2, TNFα, IL-12, IL-6, CXCL9, CXCL10 (in PTB and LTB); ↑ IL-10, TGFβ; impaired immunity may ↑ TB susceptibility	Cross-sectional (no causality), no TB incidence data, no adjustment for SES or lifestyle, gender imbalance in PTB	[79]
Prospective cohort, Eastern China, 2013–2021; 27,807 adults; median age 50; ~50% female	≤24 vs. >24 kg/m^2^; BMI as continuous variable also analysed	TB incidence (confirmed via national registry)	Age, sex, BMI, smoking, alcohol, BCG vaccination, diabetes	Lower BMI ↑ TB risk: per 1 unit ↓ BMI, aHR 0.84 (0.77–0.91); BMI ≤ 24 + diabetes → aHR 2.68 (1.32–5.43); BMI > 24 neutralised DM risk	BMI only at baseline; no HIV data; diabetes self-reported; Jiangsu-only registry—possible missed TB if moved away	[80]
Prospective cohort, South Korea, 2010–2017; 11,135,332 adults ≥20 y from NHIS screening database; 69.3% of underweight were female; mean age underweight 40.8 y	<18.5 (underweight), 18.5–22.9 (normal), 23.0–24.9 (overweight), ≥25 (obese); underweight further split: 17.0–18.4, 16.0–16.9, <16.0	TB incidence (national registry)	Age, sex, smoking, alcohol, exercise, income, hypertension, diabetes, dyslipidemia	BMI <18.5 → aHR 2.08 (2.02–2.15); mild thinness aHR 1.98, moderate 2.50, severe 2.83 vs. normal; log-linear inverse BMI–TB risk	TB type not stratified; only baseline BMI; generalizability limited to high-income settings; underweight may reflect other risks	[81]
Retrospective cohort, South Korea, 2006–2017; 2,396,434 adults; 7.27-year median follow-up; ~22% female in underweight group, younger population	BMI <18.5 (underweight); 0 to 4 accumulated episodes of underweight across 4 years	TB incidence (new diagnosis based on national registry)	Age, sex, diabetes, hypertension, dyslipidemia; stratified by sex, age, WC	TB risk ↑ with more years underweight: aHR 3.33 (3.00–3.84) for 4× underweight vs. 0×; stronger in women and those <65 years	BMI not consecutive; no HIV data; body composition not analyzed; mostly Korean subjects; no severe underweight (<16) subgroup analyzed	[82]

TB—tuberculosis; PTB—pulmonary tuberculosis; LTB—latent tuberculosis; AFB—acid-fast bacillus; BMI—body mass index; DM—diabetes mellitus; aHR—adjusted hazard ratio; OR—odds ratio; CI—confidence interval; RID—Korean National TB Registry (registration ID); SES—socioeconomic status; LBMI—low body mass index; NBMI—normal body mass index; TGFβ—transforming growth factor beta; IFNγ—interferon gamma; IL—interleukin; CXCL—CXC chemokine ligand; BCG—Bacillus Calmette–Guérin (vaccine); NHIS—National Health Insurance Service (South Korea); WC—waist circumference; FFQ—food frequency questionnaire; ↑—increase; ↓—decrease.

**Table 2:** Summary of studies investigating association between underweight or BMI and TB mortality.

Study Design and Population Characteristics	BMI Categories Analysed	Mortality Outcome	Key Findings	Confounders Adjusted For	Adherence Support	Limitations	Reference
Retrospective cohort, Taiwan, 2012–2014; 2410 adults with TB; age ≥ 18, mean age 64.5 years, 67.1% men	<18.5 (underweight), 18.5–24.9 (normal), ≥25 (overweight)	TB-specific and non-TB-specific mortality during treatment; early (<8 weeks) and late (>8 weeks); mortality: Underweight 24.2%, Normal 14.0%, Overweight 10.4%	Underweight ↑ all-cause mortality (AHR 1.57, CI 1.26–1.95), TB-specific (AHR 1.85, CI 1.03–3.33), non-TB-specific (AHR 1.52, CI 1.19–1.95); strongest effect in first 8 weeks: TB-specific AHR 2.23 (CI 1.09–4.59), non-TB-specific AHR 1.81 (CI 1.29–2.55)	Age, sex, education, marital status, unemployment, smoking, alcohol, diabetes, ESRD, malignancy, AFB smear, TB culture, CXR findings, DOT, extrapulmonary TB	Yes—DOTS (Directly Observed Treatment Short-Course) program applied to most	BMI self-reported, only baseline value; retrospective design; some clinical data not collected (e.g., IV drug use); limited generalizability beyond Taiwan	[95]
Prospective cohort, Korea, 2019–2020; 9721 patients with pulmonary TB; mean age 61.8 years, 36.5% female	Underweight < 18.5, Normal 18.5–22.9, Overweight ≥ 23 (Asian cut-offs)	All-cause, TB-related, non-TB-related mortality during anti-TB treatment; underweight mortality 19.3%, normal 10.0%, overweight 8.2%	Underweight ↑ mortality: all-cause aOR 1.95 (1.67–2.27), TB-related aOR 2.06 (1.55–2.74); Overweight protective (e.g., TB-related death aOR 0.69, 95% CI 0.48–0.99)	Age, sex, smoking, heavy alcohol, comorbidities (e.g., diabetes, heart/lung/kidney/liver disease, malignancy)	Yes—PPM program with TB nurse monitoring until treatment end	No inflammatory biomarkers; generalizability limited (low HIV prevalence, high-income country); BMI only at baseline	[96]
Retrospective cohort, Georgia, 2009–2020; 720 adults with M/XDR-TB; median age 35.5 (IQR: 26.5–49.0), 68.8% male	<18.5 (low), 18.5–24.9 (normal), ≥25 (high); BMI change during first 3–6 months categorized as negative, no change, positive	All-cause mortality during and after treatment; 16 died during (2.2%), 50 post-treatment (6.9%)	No BMI gain in low BMI group ↑ post-TB mortality (aHR 4.99, CI 1.25–19.94); weight loss in normal BMI ↑ mortality during treatment (aHR 5.25, CI 1.31–21.10)	Age, gender, year of treatment initiation, baseline sputum smear, chest X-ray cavitation	Yes—DOT program (directly observed therapy)	34.8% excluded for missing BMI follow-up; no data on severity biomarkers or treatment regimens; retrospective design limits causality	[97]
Retrospective cohort, Taipei, Taiwan, 2011–2012; 1608 adults with TB; age 18–112 (mean 64.6), 67.5% male	<18.5 (underweight), 18.5–24.9 (normal), ≥25 (overweight)	TB-specific, non-TB-specific, and all-cause mortality during treatment; underweight death rate 24.4%, normal 14.2%, overweight 10.3%	Underweight ↑ all-cause mortality (AOR 1.66, CI 1.21–2.30), TB-specific (AOR 2.14, CI 1.18–3.89), non-TB-specific (AOR 1.58, CI 1.11–2.25); effect only in males: TB-specific AOR 2.37 (CI 1.19–4.72)	Age, sex, education, unemployment, AFB smear, pleural effusion, ESRD, malignancy	Not mentioned	BMI only at baseline; 25.7% TB diagnosed clinically, possible overdiagnosis; retrospective design; missing info (e.g., IV drug use)	[98]
Prospective cohort, India, 2015–2019; 2931 adults with drug-sensitive pulmonary TB from 5 academic sites; age >18; 70.8% male	<16, 16–16.99, 17–18.49, 18.5–22.99 (ref), ≥23; also premorbid BMI (same cut-offs); BMI change after 2 months	All-cause mortality; part of composite outcome (death, treatment failure, relapse); 3.4% died	BMI < 16 at treatment start: aIRR 2.05 (1.42–2.98); premorbid BMI < 16: aIRR 2.20 (1.16–3.94); unchanged/decreased BMI after 2 months: aIRR 5.16 (1.51–17.65); severe stunting: aIRR 1.52 (1.00–2.24)	Age, sex, symptom duration, sputum smear grade, smoking, alcohol use disorder, diabetes, HIV status	Not mentioned; no objective adherence measurement	Self-reported premorbid weight (recall bias); no adherence data; variable diabetes definitions; possible residual confounding; limited generalizability outside India	[99]
Prospective cohort, India (Chennai), 2014–2018; 389 adults with drug-sensitive pulmonary TB, aged 25–60; 256 with diabetes, 133 with normal glucose tolerance	BMI < 18.5 vs. ≥18.5; HbA1c < 8.0% vs. ≥8.0% used for subgrouping; 4 groups: BMIlo/A1clo, BMIlo/A1chi, BMIhi/A1clo, BMIhi/A1chi	All-cause mortality and treatment failure (composite outcome); BMIlo/A1clo had worst outcomes	BMI < 18.5 strongest predictor of failure/death: aOR 4.99 (1.77–11.36); paradoxical protective effect of DM with HbA1c ≥ 8.0 in underweight: aOR 1.48 (0.42–5.19); VAI > 5.0: OR 13.5 (1.4–135.0) in underweight	Age, sex, income, height, smoking, alcohol use	Yes—Treatment under national TB program (not directly measured)	Small subgroups; adherence not objectively assessed; no detailed nutrition data; no vitamin A or insulin resistance markers; population-specific metabolic effects may not generalize	[100]
Retrospective cohort, India, 2004–2009; 1695 adults with pulmonary TB, rural setting; median age 38, 68% men	<16 (severe), 16–16.99 (moderate), 17–18.49 (mild), 18.5–24.9 (normal); continuous BMI also used	TB-related mortality during treatment; 60 deaths among 1179 treated (5.1%)	BMI < 16 → 2-fold ↑ TB death risk; BMI 13 → aOR 3.9 (CI 1.7–8.3); each 1 unit ↑ BMI → aOR 0.78 (CI 0.68–0.90); effect stronger in men	Age, sex, weight, height, smear status, HIV status, treatment history	Daily self-administered therapy at low cost; counselling; DOT not mentioned	Retrospective; no data on disease extent or adherence; some missing heights; limited generalizability; chronic undernutrition prevalent	[101]
Retrospective cohort, Ethiopia, 2008–2012; 810 adult TB patients; mean age 32.4, 61% EPTB, 18.3% HIV+	Body weight < 35 kg vs. ≥35 kg (BMI not used)	All-cause mortality during treatment; 60 deaths (7.4%), 56.7% in intensive phase	Weight < 35 kg → AHR 3.90 (CI 1.63–9.33); age and HIV status also significant predictors	Age, sex, type of TB, HIV status, ART use	Yes—DOTS program in all clinics	Body weight not BMI; limited data on comorbidities and MDR-TB; retrospective design; possible underreporting of causes	[102]

Legend: TB—tuberculosis; AHR—adjusted hazard ratio; CI—confidence interval; aOR—adjusted odds ratio; DOT—directly observed therapy; DOTS—Directly Observed Treatment Short-Course; CXR—chest X-ray; AFB—acid-fast bacillus; ESRD—end-stage renal disease; M/XDR-TB—multidrug/extensively drug-resistant tuberculosis; IQR—interquartile range; HbA1c—glycated haemoglobin; BMIlo/A1clo—low BMI and low HbA1c; BMIlo/A1chi—low BMI and high HbA1c; BMIhi/A1clo—high BMI and low HbA1c; BMIhi/A1chi—high BMI and high HbA1c; VAI—vitamin A index; ART—antiretroviral therapy; EPTB—extrapulmonary tuberculosis; DM—diabetes mellitus; ↑—increase; ↓—decrease.

**Table 3:** Common nutritional deficiencies in TB patients.

Nutrient	Clinical Impact	Aetiology and Mechanisms	Reference
Energy (Calories)	Weight loss, fatigue, decreased physical function	Elevated basal metabolic rate; reduced appetite; nutrient malabsorption	[119,120]
Protein	Muscle wasting, impaired immunity, delayed recovery	Increased catabolism due to systemic inflammation; anorexia leading to reduced intake; anabolic block, where dietary protein is used more for energy than for tissue synthesis	[121,122,123]
Copper	Anaemia, neutropenia, impaired immune response, neurological symptoms	Host macrophages increase copper concentration in phagosomes to combat M. tuberculosis; the bacterium counters by upregulating copper efflux pumps (e.g., CtpV) and metallothioneins (e.g., MymT), leading to disrupted host copper homeostasis and potential systemic deficiency	[119,120]
Iron	Anaemia, reduced oxygen transport	Anaemia of chronic disease due to inflammation; iron sequestration; decreased absorption	[119,124]
Selenium	Weakened antioxidant defence, increased oxidative damage	Reduced intake; increased utilisation during oxidative stress	[125]
Vitamin A	Impaired mucosal immunity, increased infection risk	Decreased intake, malabsorption, increased urinary loss, and the acute phase response reducing serum levels	[124,126,127]
Vitamin B12	Megaloblastic anaemia, peripheral neuropathy, cognitive disturbances, fatigue	Reduced dietary intake due to anorexia; malabsorption from gastrointestinal involvement	[127,128]
Vitamin C	Impaired collagen synthesis, delayed wound healing	Increased oxidative stress depletes stores; reduced intake due to anorexia	[129,130]
Vitamin D	Compromised macrophage function, increased susceptibility to infection, increased risk of disseminated TB	Limited sun exposure, decreased dietary intake, and inflammation-induced sequestration; reduced innate immunity and antimicrobial peptide synthesis; associated with more severe TB phenotypes and extrapulmonary disease in vitamin D-deficient individuals	[124]
Vitamin E	Reduced antioxidant capacity, increased cellular damage	Enhanced oxidative stress from chronic inflammation; decreased dietary intake	[124]
Zinc	Impaired immune response, delayed wound healing	Redistribution during acute phase response; decreased intake; increased losses	[119,126]
Alanine	Impaired energy metabolism; muscle wasting; fatigue and reduced exercise tolerance	Catabolised by Mtb as a nitrogen and carbon source; restored after TB therapy due to reduced Mtb demand; restored host protein metabolism	[131,132]
Glutamine	Suppressed immune response; increased oxidative stress; higher risk of secondary infections	Used by both Mtb (nitrogen source) and host immune cells (ATP and cytokines); restored after TB therapy	[131,132]
Histidine	Anaemia and fatigue; impaired antioxidant capacity; altered inflammation regulation	Used in host immune response; altered in wasting syndrome; restored after TB therapy due to restored protein metabolism and reduced inflammatory burden	[133,134]
Lysine	Reduced wound healing capacity; impaired immune response; muscle catabolism and weight loss	Rapidly metabolised as a nitrogen donor for Mtb	[131]
Tryptophan	Depression, mood disturbances, sleep disorders; immunosuppression via kynurenine pathway activation	Catabolised to kynurenine via IDO1; suppresses T-cell proliferation; restored after TB therapy due to Reduced IDO1 activity; immune reactivation	[135,136]

**Table 4:** Drug-induced nutritional deficiencies in TB therapy.

Drug	Nutritional Deficiency	Mechanism	Reference
Isoniazid	Vitamin B6	isoniazid binds to pyridoxal 5′-phosphate (active form of vitamin B6) → inactivation → depletion → impairs neurotransmitter synthesis (GABA) → risk: peripheral neuropathy and seizures	[141]
	Vitamin B3	isoniazid inhibits tryptophan → niacin conversion by interfering with vitamin B6-dependent enzymes + directly disrupts intracellular niacin synthesis → pellagra	[142]
	Vitamin D	impairs 25-hydroxylation → impaired vitamin D action	[143]
Rifampicin	Vitamin K	very rare, possibly by disrupting the vitamin K cycle	[144]
	Vitamin D	rifampin induces P450 → accelerates vitamin D metabolization into inactive forms → reduces circulating levels of 25-hydroxyvitamin D and 1,25-dihydroxyvitamin D → deficiency	[145]
Pyrazinamide	Coenzyme A	pyrazinoic acid inhibits the aspartate decarboxylase (needed for β-alanine biosynthesis, precursor of vitamin B5 → disrupts coenzyme A synthesis	[146]
Ethambutol	Zinc	ethambutol chelates zinc → reduced absorption and increased urinary excretion → to zinc deficiency → optic neuropathy.	[147]
	Copper	ethambutol binds to copper → deficiency → altered mitochondrial function and oxidative phosphorylation → optic nerve damage.	[148]
	Vitamin B1	prolonged use → decreased vitamin B1 levels → optic neuropathy.	[147]
	Vitamin E	prolonged use → decreased vitamin E levels → optic neuropathy	[147]
Cycloserine	Vitamin B6	cycloserine forms an inactive covalently bound complex with pyridoxal 5′-phosphate → a functional vitamin B6 deficiency → anaemia and peripheral neuropathy	[149]
Ethionamide	Vitamin B6	rare, neurotoxic effects	[150]
Linezolid	Vitamins B1, B9, B12	prolonged use → deficiencies → peripheral neuropathy and haematological abnormalities	[151,152]
Aminoglycosides	Magnesium, calcium, potassium	renal tubular dysfunction → increased excretion of magnesium and calcium	[153]
Clofazimine	Vitamins A, D, E, K, B9, B12	causes crystal-storing histiocytosis → damages the intestinal mucosa → villous blunting, inflammation, and loss of absorptive surface area → impaired absorption of vitamins A, D, E, K, B9, B12	[154]

**Table 5:** Food–drug interactions in TB management.

Nutrient/Food	Drug	Mechanism	Reference
Alcohol	cycloserine	neurotoxicity risk: seizures and psychosis	[157]
	isoniazid, ethionamide, PAS, pyrazinamide, rifampin, ethionamide	hepatotoxicity: additive effects on hepatic metabolism	[158]
Caffeine (coffee, tea, cola, chocolate)	isoniazid	central nervous system stimulation; isoniazid inhibits the metabolism of caffeine; heightened side effects: restlessness, insomnia, increased heart rate	[159]
Dairy products, iron supplements	fluoroquinolones	reduced absorption: calcium, magnesium, aluminium, and iron can bind to fluoroquinolones, forming insoluble complexes	[160]
Food	isoniazid, rifampicin	notable reductions in drug exposures and peak concentrations, a delay in reaching peak drug concentrations	[161]
	bedaquiline, cycloserine, ethionamide, delamanid, PAS, pretomanid	improved gastrointestinal tolerance	[162]
Histamine-rich foods (certain fish like tuna, mackerel, salmon)	isoniazid	DAO inhibition; histamine accumulation: flushing, hypotension, gastrointestinal discomfort.	[163]
Tyramine-rich foods (aged cheeses, cured meats, soy products, red wine)	isoniazid	MAO inhibition; elevated tyramine levels, resulting in headache, flushing, palpitations, and hypertension	[164]
	linezolid	Hypertensive crisis risk: hypertensive crises.	[165]

**Table 6:** Recent clinical trials with nutritional interventions in TB management.

Study Design and Population Characteristics	Nutritional Intervention	Impact on Outcome	Reference
Cluster randomized trial, Jharkhand (India); ~2800 adult TB patients and ~11,200 household contacts (HHCs); 2-year follow-up	Index patients: monthly food basket (1200 kcal, 52 g protein/day), multivitamins; HHCs in intervention arm: 750 kcal, 23 g protein/day + multivitamins; both groups received nutritional counselling	Primary: reduction in TB incidence among HHCs; Secondary: improved nutritional status, reduced infections and mortality, improved treatment adherence and performance status in patients; final outcomes pending trial completion	[182]
Double-blind placebo-controlled RCT, China (Weifang); 329 adults (aged 18–80) with pulmonary TB + prediabetes or diabetes; 6-month follow-up	Daily nutrition package (112 kcal, 9.1 g protein, macro + 13 vitamins/minerals incl. A, B1, B2, B6, B12, C, D, E, folate, niacin, iron, Ca, Zn); vs. placebo (same calories, 3 g protein, no micronutrients)	↓ Chest pain, expectoration, and anaemia; ↑ haemoglobin, albumin, lymphocyte count; no overall sputum conversion benefit, but faster in non-cavitary TB; no significant weight change; no major adverse effects	[183]
Randomized pilot implementation study, Senegal (Ziguinchor and Bignona); 26 HIV-TB co-infected adults; 6-month follow-up; median age 46 y; 50% female	Monthly food basket (local cowpeas, rice, oil, milk: ~1200 kcal/day) or RUTF (Plumpy’Nut, ~1000 kcal/day); both arms 6 months; adherence, food security, and clinical outcomes tracked	100% TB treatment completion; all smear-negative at end; ↑ CD4 (207 → 321), ↑ Hb (10.2 → 12.8), ↑ weight (50 → 55 kg), ↑ BMI (17.3 → 19.3); ↓ food insecurity (92% → 73%); adherence to ART and TB >95% in both arms; food basket more acceptable/shared	[184]
Single-blinded RCT, Pakistan (PIMS TB centre), 2020–2021; 426 adult TB patients; 213 intervention, 213 control; follow-up at 3 and 6 months	Pharmacist-led patient-centred care: individualised counselling, printed materials on nutrition and drug use, lifestyle education, medication management, SMS and phone reminders; all patients continued standard TB therapy	EQ-5D utility score ↑ from 0.40 to 0.89 (vs. 0.42 to 0.78 in control); significant improvement in HRQoL domains (mobility, self-care, activities, pain, anxiety); ↑ patient satisfaction and adherence indicators	[185]

Legend: TB—tuberculosis; HHCs—household contacts; kcal—kilocalories; g—grams; aHR—adjusted hazard ratio; aIRR—adjusted incidence rate ratio; RCT—randomized controlled trial; Ca—calcium; Zn—zinc; Hb—haemoglobin; BMI—body mass index; CD4—CD4+ T lymphocyte count; RUTF—ready-to-use therapeutic food; ART—antiretroviral therapy; HRQoL—health-related quality of life; PIMS—Pakistan Institute of Medical Sciences; SMS—short message service; ↑—increase; ↓—decrease.
